# Dual targeting of MEK and PI3K effectively controls the proliferation of human EGFR-TKI resistant non-small cell lung carcinoma cell lines with different genetic backgrounds

**DOI:** 10.1186/s12890-021-01571-x

**Published:** 2021-07-01

**Authors:** Ge-Ping Qu, Min Shi, Dan Wang, Jiong-He Wu, Peng Wang, Mei-Liang Gong, Zhi-Jian Zhang

**Affiliations:** 1 Department of Respiratory and Critical Care Medicine, Second Medical Center of Chinese PLA General Hospital, No. 28 of Fuxing Street, Haidian District, Beijing, 100853 China; 2 Department of Laboratory Diagnosis, Second Medical Center of Chinese, PLA General Hospital, Beijing, 100853 China

**Keywords:** Non-small cell lung cancer, Tyrosine kinase inhibitor, MEK, PI3K, EGFR, MET

## Abstract

**Background:**

Molecular targeted therapy for non-small cell lung carcinoma (NSCLC) is restricted due to resistance to epidermal growth factor receptor (EGFR) tyrosine kinase inhibitors (TKIs). This study evaluated the effects of dual targeting of MEK and PI3K in human EGFR-TKI resistant NSCLC cell lines.

**Methods:**

EGFR-TKI resistant NSCLC cell lines H1975, H460, and A549, with different mutation and amplification status in *EGFR*, *K-RAS*, *PIK3CA*, and *MET* genes, were treated with a MEK162 (MEK inhibitor) and BKM120 (PI3K inhibitor) combination or a BIBW2992 (EGFR inhibitor) and ARQ197 (MET inhibitor) combination and assayed for cell proliferation, apoptosis, and cell cycle distribution.

**Results:**

Dual targeting of MEK and PI3K efficiently inhibited the cell proliferation, induced apoptosis and the G0/G1 cell cycle, and decreased the phosphorylation of ERK1/2, AKT, S6, and 4E-BP1. H460 cells with *K-RAS* and *PIK3CA* mutation were most sensitive to MEK162 and BKM120 combinations. H1975 cells with EGFR and PIK3CA mutation and MET amplification were sensitive to BIBW2992 and ARQ197 combinations.

**Conclusion:**

Dual targeting regulated the proliferation of EGFR-TKI-resistant NSCLC cells, especially mutants in K-RAS and PIK3CA that are promising for EGFR-TKI-resistant NSCLC therapeutics.

## Background

The epidermal growth factor receptor (EGFR) family of receptor tyrosine kinases has been indicated to play a significant role in the pathogenesis and progression of tumorigenesis [1]. Targeted therapies involving the use of EGFR tyrosine kinase inhibitor (TKI) as a first-line of therapeutic agents in patients with non-small cell lung cancer (NSCLC) with EGFR activating mutations (exon 19 deletions and L858R point mutations) provides superior efficacy, presents dramatic improvement in the progression-free survival, and offers negligible toxicity in comparison to other chemotherapeutics [2, 3]. However, resistance to the EGFR-TKI markedly limits its clinical use [4, 5].

The most important primary mechanism of resistance is the K-RAS mutation, which is mutually exclusive to EGFR activating mutations [6]. In the case of acquired EGFR-TKI resistance, common mechanisms include the emergence of an EGFR gatekeeper mutation (T790M) and *MET* gene amplification [7, 8]. In addition, PIK3CA mutations can contribute toward EGFR-TKIs resistance in a subpopulation of NSCLC tumors [9]. However, in either primary or acquired resistance, the downstream pathways involving RAS/RAF/MAP kinase-ERK kinase (MEK)/extracellular-signal-regulated kinase (ERK) pathway and the PI3K/protein kinase B (PKB/AKT)/mammalian target of rapamycin (mTOR) pathway are constitutively activated. This leads to continuous malignant proliferation and metastasis [10–12]. Frequently, the MEK and PI3K signaling pathways are abnormally activated in NSCLC, thus, rendering them promising candidates for the development of potential molecular targeting agents in therapies for NSCLC [13–15].

A significant amount of cross-talk occurs between the MEK and PI3K pathways to provide compensatory signaling when either is inhibited [16–18]. Therefore, therapeutic strategies to block either the MEK or PI3K pathway are not sufficient to inhibit the growth of NSCLC cells, providing the rationale for combining therapeutic agents that could simultaneously block both pathways [19]. Similar compensatory cross-talk is observed between the EGFR and MET pathways [20, 21]. We have previously confirmed that the efficacy of the combined inhibition of EGFR and MET pathways is biologically higher than the inhibition of either pathway in controlling erlotinib-resistant NSCLC cells with the EGFR T790M mutation [22].

MEK162 (Binimetinib; ARRY-162 or ARRY-438162) is an orally administered non-ATP-competitive allosteric inhibitor of MEK1/2 [23]. BKM120 (Buparlisib) is an oral pan-PI3K inhibitor against both wild-type and mutant class I PI3K isoforms [12]. However, in some studies, inhibition of MEK signaling alone with MEK162 is inadequate in treating EGFR-TKI resistant NSCLC cells, and negative feedback mechanisms in the PI3K pathway may be challenging when used independently [24]. In contrast, a combined blockade of both pathways with MEK162 and BKM120 overcame the reciprocal pathway activation and resulted in a significant tumor growth inhibition [17, 19, 25]. Clinical trials of such combinations are underway in solid tumors, including NSCLC with K-RAS mutations [25, 26]. However, the mechanisms of dual MEK and PI3K inhibition in the treatment of NSCLC cells, especially in different mutation statuses of the NSCLC, have not been extensively examined. The clinical impact of this parallel pathway blockade is not clear. Therefore, in the current study, we evaluated the efficacy of the MEK162/BKM120 combination to inhibit MEK and PI3K pathways and analyzed the effect on cell proliferation, apoptosis, and cell cycle distribution in a panel of three different human NSCLC cell lines selected according to their mutation and amplification status for *EGFR*, *MET*, *KRAS*, and *PIK3CA* genes. The data will further assist in developing molecular targeted therapeutic strategies against EGFR-TKI resistant NSCLC.

## Methods

### Cell culture

Human lung adenocarcinoma cell lines H1975, H460, and A549, were purchased from the Cell Biology Institute of the Chinese Academy of Sciences, Beijing, China. The H1975 cell line harbors the EGFR T790M mutation accompanied with MET gene amplification. The H460 cell line carries a mutation in K-RAS and PIK3CA. The A549 cell line carries the K-RAS mutation. None of the three cell lines were sensitive to first-generation EGFR-TKI [27, 28].

The cell lines were cultured in RPMI-1640 (Invitrogen, Carlsbad, CA, USA) supplemented with 10% FBS (Invitrogen), 100 units/mL penicillin (Jingmei Biotechnology Co. Ltd., Shanghai, China), and 100 mg/mL streptomycin (Jingmei Biotechnology) at 37 °C in a humidified environment containing 5% CO_2_.

### Cell treatment

H1975, H460, and A549 cells were treated with 0–3 μM MEK162 (MEK inhibitor, Sigma, St. Louis, MO, USA) or 0–15 μM BKM120 (PI3K inhibitor, Sigma), alone or in combination for 48 h. H1975, H460, and A549 cells were treated with 1 μM BIBW2992 (EGFR inhibitor, Sigma), a second-generation EGFR-TKI, or 2 μM ARQ197 (MET inhibitor, Sigma) combination for 48 h. A stock solution of the chemical inhibitors was prepared in 100% DMSO and further diluted with normal saline. The inhibitors were used at a final concentration of 0.05% DMSO.

### Cell viability assay

A colorimetric assay, based on the MTT dye [3-(4,5-dimethylthiazol-2-yl)-2,5-diphenyltetrazolium bromide] reduction was employed to assess the cell viability [29]. The previously described procedure was adopted [22]. Briefly, the cell lines (density, 1 × 10^4^ cells/well) were cultured in 96-well plates for 16–20 h. After treatment for 48 h with the indicated compounds, the media was replaced by 0.5 mg/mL MTT (Jingmei Biotechnology) in complete medium for four hours. Post incubation, the MTT containing medium was replaced with 150 μL DMSO. The absorbance was measured at 565 nm using an OptiMax plate reader (GE Healthcare, Shanghai, China), and the viability was expressed as a percentage relative to the control cells.

### Apoptosis assay

A flow cytometry-based assay for the determination of apoptosis, over a period, in cells treated with the indicated compounds was adopted as described previously [22]. The annexin V-fluorescein isothiocyanate (FITC) and propidium iodide (PI) [15] double staining apoptosis detection kit was procured from Jingmei Biotechnology Co. Ltd. Briefly, 48 h post-treatment of 1 × 10^6^ cells with the indicated compounds, the cells were washed twice with a phosphate-buffered solution (PBS; pH 7.4), and resuspended in 500 μL binding buffer before adding 5 μL annexin V and 1 mg/mL PI. The samples were examined on an LSR flow cytometer (BD Biosciences, San Jose, CA, USA) and analyzed using the CellQuest software (BD Biosciences). The early apoptotic cells were stained positive for annexin V and negative for PI, while the late apoptotic cells were positive for both annexin V and PI.

### Analysis of cell cycle distribution

Cells (1 × 10^6^) were cultured in p60 Petri dishes for 16–20 h and treated with the indicated compounds for an additional 48 h. Post incubation, the cells were fixed overnight at − 20˚C with 70% ethanol. The cells were harvested and stained with 4 mg/mL PI containing 1% Triton X-100 and 100 mg/mL RNase A for 30 min. The stained cells were passed through nylon membranes (BD Biosciences), examined on an LSR flow cytometer, and analyzed by CellQuest software. The DNA content (1 × 10^4^ cells/sample) and the percentage distribution of cells in different cell cycle phases (G0/G1, S, G2/M) were analyzed using the ModFit LT ver. 3.0 (BD Biosciences) software.

### Western blot analysis

The cellular protein samples for Western blot analysis were prepared as previously described [22]. Equal protein amounts (50–100 μg/lane) were subjected to 12% SDS–polyacrylamide gel electrophoresis and transferred electrophoretically to nitrocellulose membranes. The membranes were incubated with rabbit anti-human EGFR, p-EGFR (Y1068), MET, and p-MET (Y1234/1235) purchased from Santa Cruz Biotechnology (Santa Cruz, CA, USA), rabbit anti-human AKT, p-AKT (S473), ERK1/2, p-ERK1/2 (T202/Y204), and β-actin antibodies from Abcam (Cambridge, UK). The binding of primary antibodies was detected using goat anti-rabbit secondary antibody conjugated to horseradish peroxidase (Cell Signaling Technology, Beverly, MA, USA). The immunodetection was performed using an enhanced chemiluminescence kit (ECL; Pierce, IL, USA). The membranes were exposed to X-ray film and imaged.

### Statistical analysis

All data were presented as means ± standard deviation (SD) and were analyzed by the Student's *t*-test or one-way analysis of variance (ANOVA) with least significant difference (LSD) as post hoc test for comparison between multiple groups using Statistics Package for Social Science (SPSS) software (version 13.0; SPSS, Chicago, IL, USA). The significance was determined at *P* < 0.05.

## Results

### MEK162/BKM120 combination inhibits the viability of human EGFR-TKI resistant NSCLC cell lines with different genetic backgrounds

**Fig. 1:**
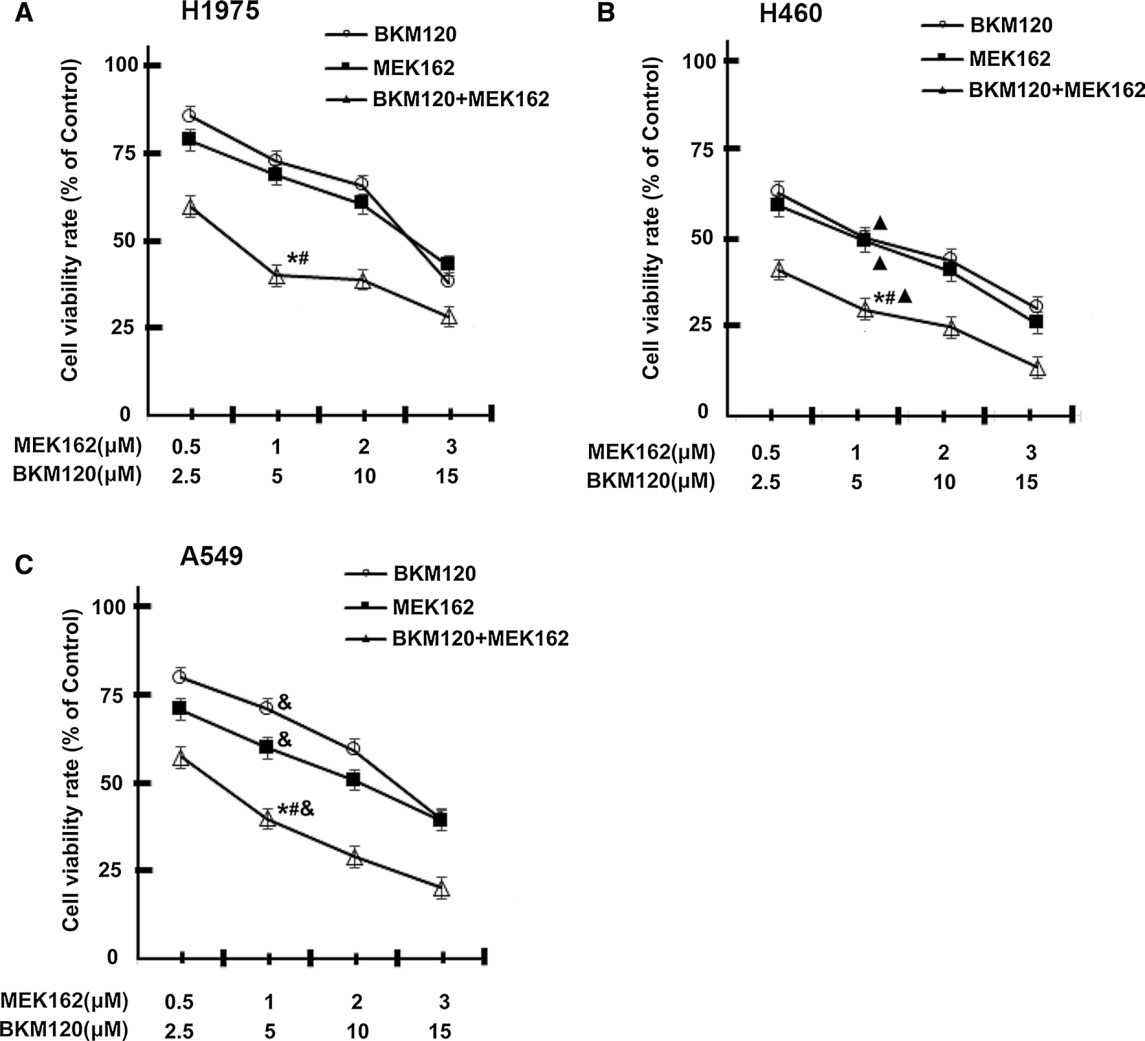
Effect of MEK162/BKM120 combination therapy on human non-small cell lung carcinoma (NSCLC) H1975, H460, and A549 cell viability. H1975 (**A**), H460 (**B**), and A549 (**C**) cells were treated with MEK162 (MEK inhibitor) (0–3 µM) or BKM120 (PI3K inhibitor) (0–15 µM) alone or their combination for 48 h, and cell viability was evaluated by MTT assay. Data are the percentages of viable cells compared with the untreated controls (0.05% DMSO) and are represented as means ± standard deviation (SD) from three independent experiments. *P < 0.05 vs. 1 μM MEK162; ^#^*P* < 0.05 vs. 5 μM BKM120; ▲*P* < 0.05 vs. H1975; &*P* < 0.05 vs. H460

H1975, H460, and A549 cells were treated with MEK162 (0–3 μM) or BKM120 (0–15 μM) or in combination for 48 h. Post incubation, the effect on cell viability was evaluated by MTT assay and described in Fig. 1. The combination of 1 µM MEK162 and 5 µM BKM120 significantly inhibited the growth of all three cell lines tested, compared with MEK162 (1 µM) or BKM120 (5 µM) alone (all *P* < 0.05). The growth inhibitory effects of 1 µM MEK162/5 µM BKM120 combination were significantly higher in H460 cells as compared to the effects in H1975 and A549 cells (both *P* < 0.05). For instance, only 13.8% viability was observed for H460 cells treated with the combination of 3 μM MEK162 and 15 μM BKM120 for 48 h. However, 26.3% and 28.5% viable cells could be detected in H1975 cells and A549 cells, respectively, after the same treatment.

### MEK162/BKM120 combination induces apoptosis in human EGFR-TKI resistant NSCLC cell lines with different genetic backgrounds

**Fig. 2:**
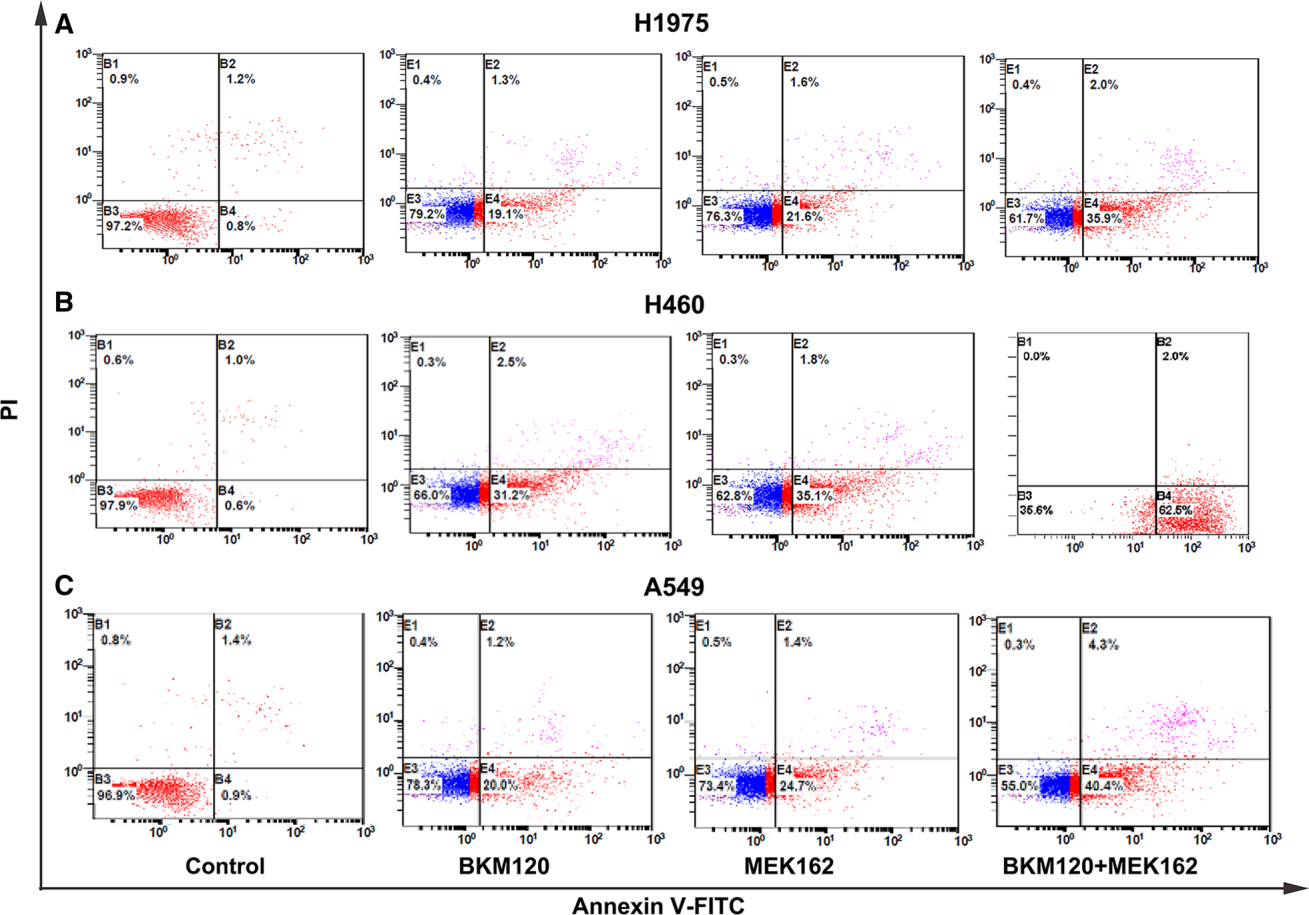
Effect of MEK162/BKM120 combination therapy on H1975, H460, and A549 cell apoptosis. H1975 (**A**), H460 (**B**), and A549 (**C**) cells were treated with 1 µM MEK162 or 5 µM BKM120 alone or their combination for 48 h. Control cells were treated with 0.05% DMSO. Cells were simultaneously stained with annexin V-fluorescein isothiocyanate (FITC) and propidium iodide (PI), and the apoptosis rates were assessed by flow cytometry. Cells positive for annexin V-FITC and negative for PI were in early apoptosis

H1975, H460, and A549 cells were treated with 1 μM MEK162 or 5 μM BKM120 or in combination for 48 h. Apoptosis was assessed by annexin V-PI double staining. Annexin V can be used to detect the externalization of phosphatidylserine during the progression of apoptosis; therefore, cells in early apoptosis can be identified. The rate of apoptosis was determined by flow cytometry and described in Fig. 2. Treatment with MEK162 or BKM120 induced a moderate increase in early apoptosis rate compared with the control in all three tested cell lines. However, MEK162 and BKM120 in combination, produce a significant increase in the early apoptosis rate compared with the control. The early apoptosis rate in H460 cells after MEK162 or BKM120 alone and in combination treatment were 31%, 35%, and 63%, respectively. This was significantly higher compared to H1975 cells (19%, 22%, and 36%, respectively) and A549 cells (20%, 25%, and 40%, respectively). Among the three cell lines, H460 was more sensitive to MEK162 or BKM120 alone and in combination, compared to H1975 and A549 cells.

### MEK162/BKM120 combination induces G0/G1 cell cycle arrest in human EGFR-TKI resistant NSCLC cell lines with different genetic backgrounds

H1975, H460, and A549 cells were treated with 1 μM MEK162 or 5 μM BKM120 or in combination for 48 h, and the percentages of cells in the cell cycle phases were assessed by flow cytometry. The data is summarized in Table 1. Treatment with MEK162 or BKM120 caused a moderate increase in the number of cells in the G0/G1 phase in the three cell lines. However, the treatment with MEK162 and BKM120 in combination caused a significant increase in the number of cells in the G0/G1 phase in all three cell lines (all *P* < 0.01). The increase in the G0/G1 phase was significantly higher in H460 cells as compared to H1975 and A549 cells after treatment with MEK162 or BKM120 or in combination (all *P* < 0.05). However, the proportion of the G0/G1 phase was not significantly different between H1975 cells and A549 cells (*P* > 0.05).

**Table 1:** Effect of MEK162/BKM120 combination therapy on human non-small cell lung carcinoma (NSCLC) H1975, H460, and A549 cell cycle distribution

Group	Cell cycle distribution (%)		
	G_0_/G_1_	S	G_2_/M
*H1975 cells*			
Control	60.3 ± 3.6	31.5 ± 4.8	8.2 ± 5.5
BKM120	78.4 ± 3.5*	18.9 ± 8.4	2.7 ± 1.8
MEK162	72.6 ± 7.7*	21.1 ± 6.8	6.3 ± 2.3
BKM120 ± MEK162	85.7 ± 5.5**^##@@^	10.6 ± 2.1	3.7 ± 2.4
*H460 cells*			
Control	55.9 ± 4.6	33.5 ± 4.8	10.6 ± 5.9
BKM120	72.4 ± 2.5*^▲^	24.9 ± 2.4	2.7 ± 1.1
MEK162	74.6 ± 5.4*^▲^	20.3 ± 6.8	5.1 ± 1.4
BKM120 ± MEK162	91.8 ± 6.9**^##@@▲^	6.6 ± 2.1	1.6 ± 0.8
*A549 cells*			
Control	62.8 + 3.5	26.5 + 6.8	10.7 + 5.1
BKM120	76.4 + 5.5*^&^	18.9 + 8.4	4.7 + 4.8
MEK162	77.9 + 6.7*^&^	15.8 + 3.8	6.3 + 2.9
BKM120 + MEK162	88.6 + 6.6**^##@@&^	9.2 + 3.8	2.2 + 1.2

H1975, H460, and A549 cells were treated with 1 μM MEK162 (MEK inhibitor) or 5 μM BKM120 (PI3K inhibitor) alone or in combination for 48 h. Control cells were treated with DMSO (0.05%). After treatment, the cells were stained with propidium iodide (PI) and analyzed by flow cytometry. Each value represents mean ± standard deviation (SD) obtained from three independent experiments

*P < 0.05 vs. Control; ^#^*P* < 0.05 vs. BKM120; ^@^*P* < 0.05 vs. MEK162; ^▲^*P* < 0.05 vs. H1975 cells; ^&^*P* < 0.05 vs. H460 cells under the same cell cycle phase and the same drug treatment

### MEK162/BKM120 combination regulates MEK and PI3K signaling transduction pathways of human EGFR-TKI resistant NSCLC cell lines with different genetic backgrounds

**Fig. 3:**
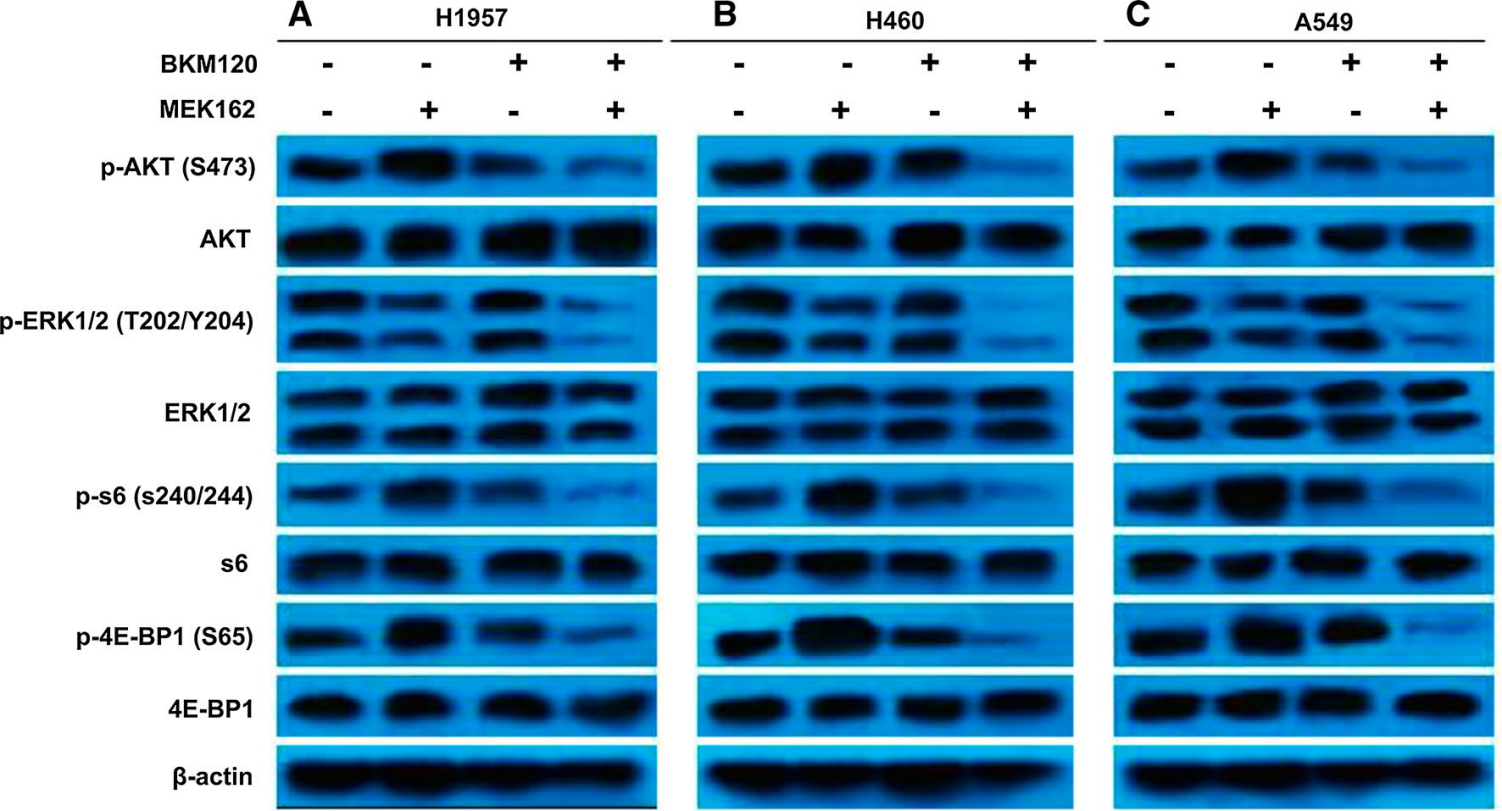
Effect of MEK162/BKM120 combination on MAPK and PI3K downstream effectors. H1975 (**A**), H460 (**B**), and A549 (**C**) cells were treated with 1 μM MEK162 or 5 μM BKM120 alone or their combination for 48 h. Control cells were treated with 0.05% DMSO. Whole cell lysates were collected for Western blot analysis. β-actin protein levels were evaluated as loading controls

H1975, H460, and A549 cells were treated with 1 μM MEK162 or 5 μM BKM120, or in combination, for 48 h. The samples were subjected to Western blot analysis to analyze the phosphorylation status and total protein expression post-treatment to assess the effect of treatment on downstream signaling molecules of the MEK and PI3K pathways (Fig. 3). An increase in the level of p-AKT and its downstream substrates p-S6 and p-4E-BP1 was detected in all three cell lines treated with MEK162 alone, but not in cells exposed to the combination of MEK162 and BKM120. In addition, the MEK162 and BKM120 combination treatment exhibited inhibitory effects on p-ERK1/2, p-AKT, p-S6, and p-4E-BP1 in comparison to the control group or cells treated with either MEK162 or BKM120 in the three cell lines, whereas the total protein levels of ERK1/2, AKT, S6, and 4E-BP1 remained unaltered in each of the cell lines. However, the effect of the MEK162/BKM120 combination on the down-regulation of p-ERK1/2, p-AKT, p-S6, and p-4E-BP1 in H460 cells was more profound compared to H1975 cells and A549 cells.

### Effects of MEK/PI3K dual inhibition or EGFR/MET dual inhibition on cell viability, apoptosis, cell cycle distribution in human EGFR-TKI resistant NSCLC cell lines with different genetic backgrounds

We have previously confirmed that dual inhibition of EGFR and MET, rather than inhibition of either of the pathways, was effective against erlotinib-resistant H1975 cells with the EGFR-T790M mutation [22]. To compare the difference of cytotoxic effects of dual upstream inhibition of EGFR/MET and dual downstream inhibition of MAPK/PI3K, we treated all the three aforementioned cell lines with a combination of 1 μM BIBW2992 (second-generation EGFR-TKI) and 2 μM ARQ197 (MET inhibitor) and a combination of 1 μM MEK162 and 5 μM BKM120 for 48 h. The samples were subjected to cell viability assay, apoptosis assay, cell cycle analysis, and Western blot analysis.

**Fig. 4:**
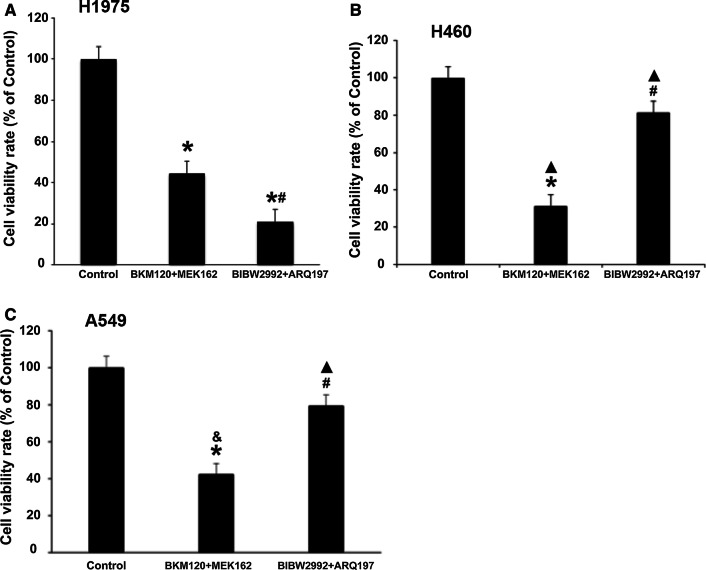
Effect of MEK162/BKM120 combination and BIBW2997/ARQ197 combination therapy on cell viability. H1975 (**A**), H460 (**B**), and A549 (**C**) cells were treated with 1 μM MEK162/5 μM BKM120 combination and 1 µM BIBW2992 (EGFR inhibitor)/2 µM ARQ 197 (MET inhibitor) combination for 48 h. Control cells were treated with 0.05% DMSO. Cell viability was evaluated by MTT assay. Data are percentages of viable cells compared with the controls and are represented as means ± SD from three independent experiments. *P < 0.05 vs. control (0.05% DMSO); ^#^*P* < 0.05 vs. BKM120 + MEK162; ▲*P* < 0.05 vs. H1975; &*P* < 0.05 vs. H460

The viability of H1975 cells treated with a MEK162/BKM120 combination was significantly higher in comparison to treatment with a BIBW2992/ARQ197 combination (*P* < 0.05) (Fig. 4A). However, significantly higher viability was observed for H460 cells and A459 cells treated with the BIBW2992/ARQ197 combination in comparison to the treatment with the MEK162/BKM120 combination (*P* < 0.05) (Fig. 4B, C). In addition, the anti-proliferative effect of the MEK162/BKM120 combination was significantly higher (both *P* < 0.05) in H460 than in H1975 and A549 cells, and the anti-proliferative effect of the BIBW2992/ARQ197 combination was significantly higher (both *P* < 0.05) in H1975 than in H460 and A549 cells (Fig. 4).

**Fig. 5:**
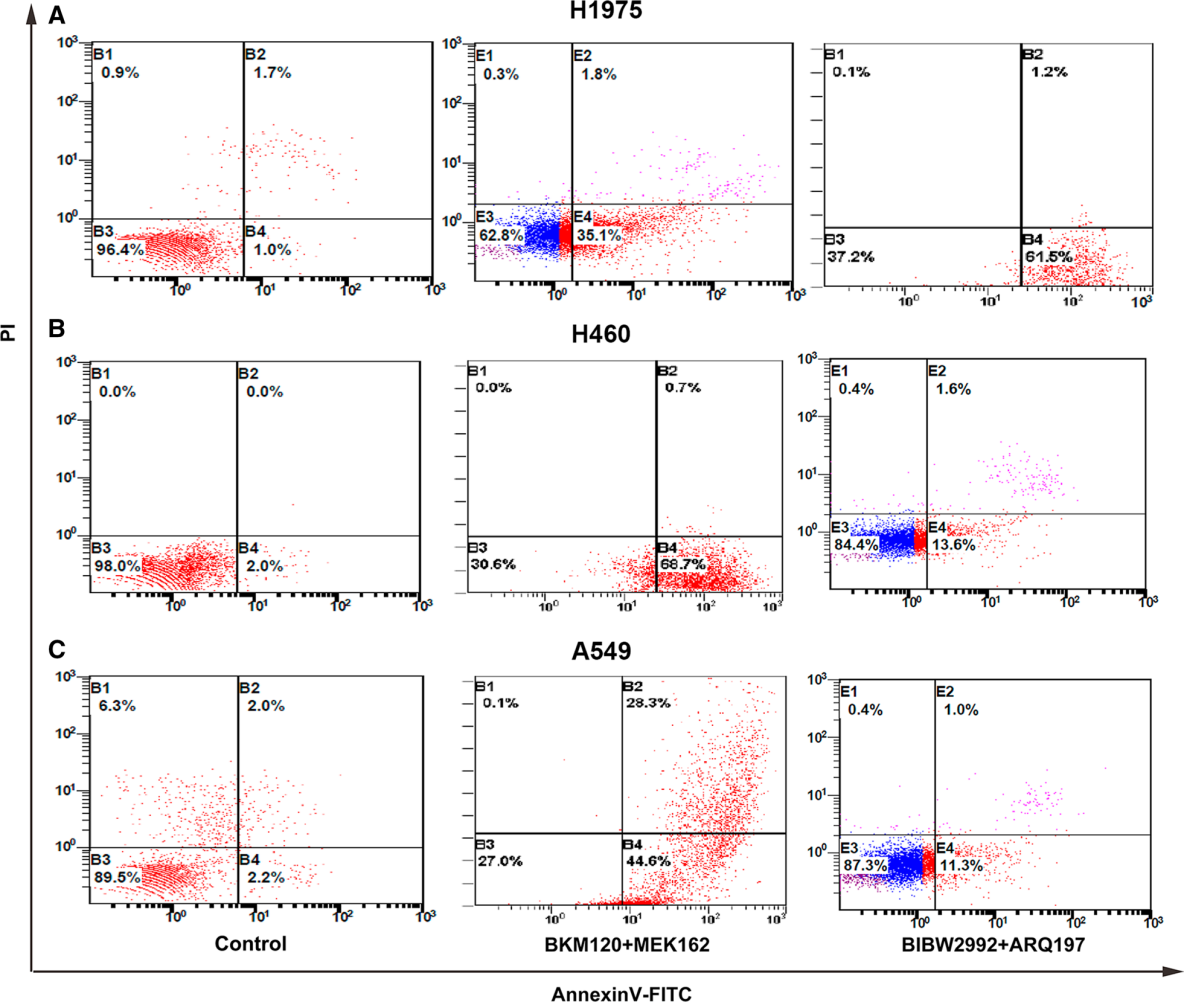
Effect of MEK162/BKM120 combination and BIBW2997/ARQ197 combination therapy on cell apoptosis. H1975 (**A**), H460 (**B**), and A549 (**C**) cells were treated with 1 μM MEK162/5 μM BKM120 combination and 1 µM BIBW2992/2 µM ARQ 197 combination for 48 h. Control cells were treated with 0.05% DMSO. Cells were simultaneously stained with annexin V- FITC and PI, and the apoptosis rates were assessed by flow cytometry. Cells positive for annexin V-FITC and negative for PI were in early apoptosis

The early apoptosis rate in H1975 cells was significantly higher for treatment with the BIBW2992/ARQ197 combination than treatment with the MEK162/BKM120 combination. However, the early apoptosis rate in H460 and A549 cells was significantly higher for treatment with the MEK162/BKM120 combination than the treatment with the BIBW2992/ARQ197 combination (Fig. 5). In addition, the rate of early apoptosis was significantly higher for H460 cells treated with the MEK162/BKM120 combination as compared to A549 and H1975 cells, and the rate of early apoptosis was significantly higher for H1975 cells treated with the BIBW2992/ARQ197 combination as compared to A549 and H460 cells (Fig. 5).

Treatment of H1975 cells with the BIBW2992/ARQ197 combination had a significantly higher proportion of cells in the G0/G1 phase compared to the treatment with the MEK162/BKM120 combination (*P* < 0.05) (Table 2). However, in a similar analysis, treatment of H460 and A549 cells with the MEK162/BKM120 combination exhibited a significantly higher proportion of cells in the G0/G1 phase than the BIBW2992/ARQ197 treatment combination (both *P* < 0.05). In addition, the G0/G1 cell cycle arrest induced by the MEK162/BKM120 combination was significantly profound in H460 cells compared to A549 and H1975 cells (both *P* < 0.05). The G0/G1 cell cycle arrest induced by the BIBW2992/ARQ197 combination was significantly profound in H1975 cells compared to A549 and H460 cells (both *P* < 0.05) (Table 2).

**Table 2:** Effect of MEK162/BKM120 combination and BIBW2997/ARQ197 combination therapy on NSCLC H1975, H460, and A549 cell cycle distribution

Group	Cell cycle distribution (%)		
	G_0_/G_1_	S	G_2_/M
*H1975 cells*			
Control	61.5 + 3.6	29.4 + 2.3	9.1 + 6.3
BKM120 + MEK162	81.3 + 2.7*	12.9 + 6.5	5.8 + 2.2
BIBW2992 + ARQ197	92.6 + 5.5*^#^	3.5 + 2.4	3.9 + 1.4
*H460 cells*			
Control	58.4 + 6.6	24.4 + 8.3	17.2 + 7.3
BKM120 + MEK162	90.5 + 3.8*^▲^	6.1 + 5.2	3.4 + 3.2
BIBW2992 + ARQ197	59.5 + 5.6^#▲^	22.7 + 3.4	17.8 + 7.4
*A549 cells*			
Control	63.1 + 9.1	25.7 + 6.3	11.2 + 4.3
BKM120 + MEK162	85.3 + 7.6*^&^	8.8 + 2.5	5.9 + 3.8
BIBW2992 + ARQ197	61.4 + 5.5^#▲^	27.1 + 6.4	11.5 + 6.4

H1975, H460, and A549 cells were treated with 1 μM MEK162/5 μM BKM120 combination and 1 µM BIBW2992 (EGFR inhibitor)/2 µM ARQ 197 (MET inhibitor) combination for 48 h. Control cells were treated with 0.05% DMSO. Each value represents mean ± SD obtained from three independent experiments

^*^P < 0.05 vs. Control; ^#^*P* < 0.05 vs. BKM120 + MEK162; ^▲^*P* < 0.05 vs. H1975 cells; ^&^*P* < 0.05 vs. H460 cells under the same cell cycle phase and the same drug treatment

**Fig. 6:**
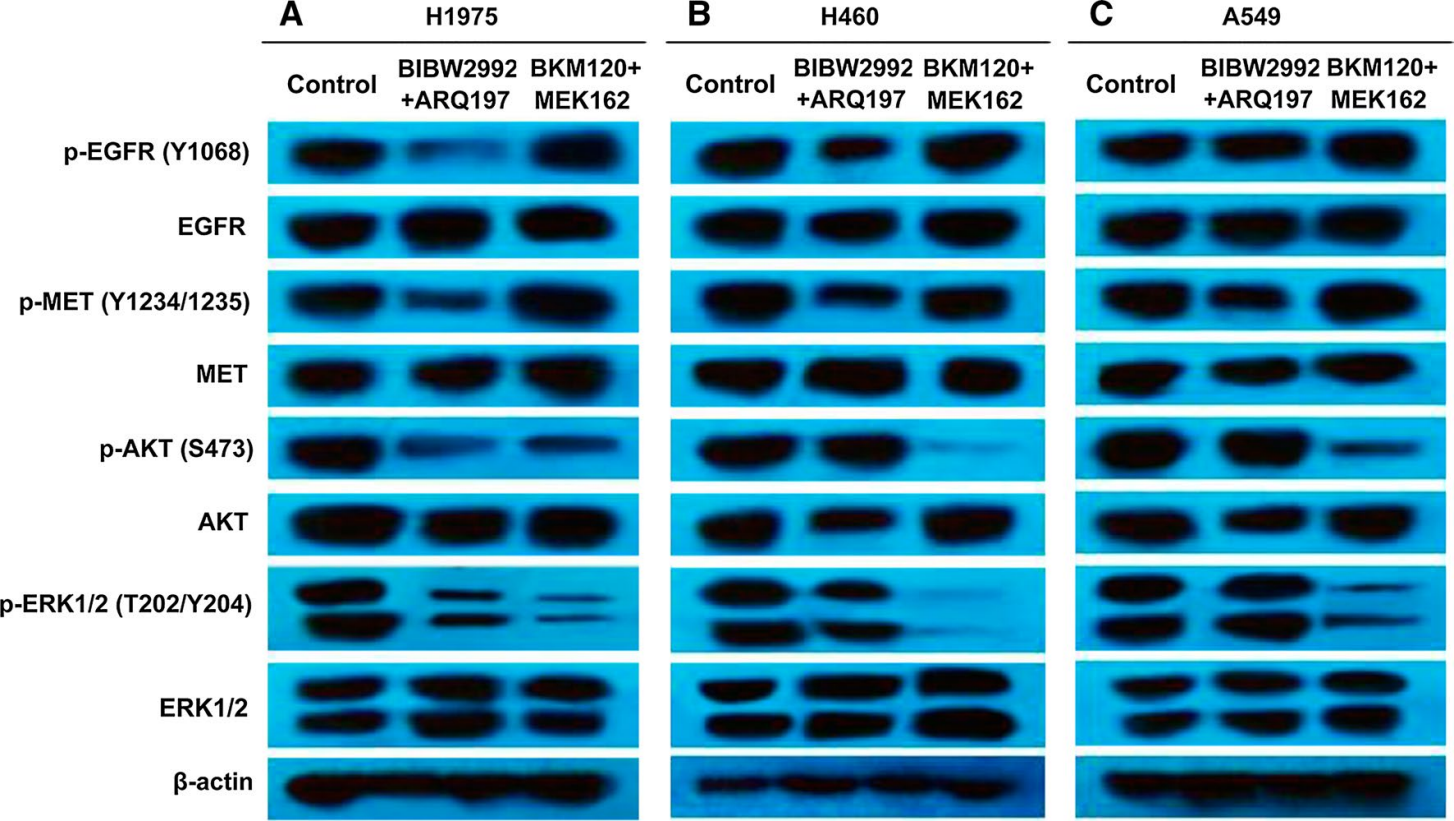
Effects of MEK162/BKM120 combination and BIBW2997/ARQ197 combination therapy on EGFR, MET, MAPK, and PI3K pathways. H1975 (**A**), H460 (**B**), and A549 (**C**) cells were treated with 1 μM MEK162/5 μM BKM120 combination and 1 µM BIBW2992/2 µM ARQ197 combination for 48 h. Control cells were treated with 0.05% DMSO. Whole cell lysates were collected for Western blot analysis. β-actin protein levels were evaluated as loading controls

H1975 cells treated with the BIBW2992/ARQ197 combination exhibited lower levels of p-EGFR and p-MET when compared to the similar treatment of H460 cells and A549 cells (Fig. 6). However, treatment of H460 cells with the MEK162/BKM120 combination exhibited lower levels of p-AKT and p-ERK compared to the similar treatment of H1975 cells and A549 cells (Fig. 6).

## Discussion

MEK and PI3K pathways are the two essential kinase cascades frequently dysregulated in various cancers, including human lung cancer [17, 19, 30, 31]. Both the pathways represent important signal transduction mechanisms that facilitate the proliferation and survival of cancers driven by growth factor receptors, such as EGFR. In the present study, we confirmed that treatment with a MEK162/BKM120 combination effectively inhibited cell proliferation, induced apoptosis, and G0/G1 cell cycle arrest in EGFR-TKI resistant NSCLC cell lines: H1975, H460, and A549. However, the treatment of cells with MEK162 or BKM120 alone was ineffective. The cytotoxic effects of the combination treatment can be attributed to the inhibition of both the MEK and PI3K pathways. Increasing evidence demonstrates that the MEK and PI3K signaling pathways may interact to promote the growth and survival of tumor cells [16, 32]. Also, EGFR activates MET through MAPK [33]. Therefore, the inefficacy of treatment with MEK162 or BKM120 alone can be attributed to the compensating cross-talk between the pathways. We also detected that the treatment with MEK162 elevated the phosphorylation of AKT in all three tested cell lines, which can be abrogated by a MEK162/BKM120 combination. In addition, a MEK162/BKM120 combination caused a decrease in the levels of p-ERK1/2, p-AKT, p-S6, and p-4E-BP1 in all three cell lines. Collectively, our data suggested that the synergistic inhibition of MEK and PI3K pathways, rather than selective inhibition of each pathway, achieved sufficient cytotoxic effects in EGFR-TKI resistant NSCLC cell lines, H1975, H460, and A549. These results corroborate the observation by Yao et al. [27] and Qu et al. [28].

It has been reported that in the MAPK pathway, activated RAF phosphorylates MEK, a dual-specificity tyrosine, and serine/threonine kinase, which predominantly activates the serine/threonine kinases, ERK1 and ERK2. ERKs are known to have key roles in controlling cell cycle regulation, proliferation, and survival [34, 35]. In the PI3K pathway, activated PI3K ultimately leads to the stimulation of AKT, which promotes cell survival by phosphorylating MDM2 (a negative regulator of the p53 tumor suppressor) and negatively regulating the pro-apoptotic Bcl-2 family members, BAD and BAX, and forkhead transcription factors, such as forkhead box O (FOXO). Activated AKT also negatively regulates the tuberous sclerosis protein complex 1 (TSC1) and TSC2, which leads to the activation of mTOR complex 1 (mTORC1), a key regulator of cellular growth and protein synthesis. MAPK pathway regulates mTOR signaling through the inactivation of the TSC1/TSC2 heterodimeric complex by ERK [35, 36]. In addition, a recent study demonstrated that inhibition of mTORC1 with everolimus leads to the activation of the MAPK pathway [36]. This phenomenon may be attributed to the theory that mTORC1 inhibition releases the "brake" on PI3K triggered by S6K, which activates the MAPK signaling. Therefore, we firmly speculate that the interactions between the two signaling pathways underlie the need to block both these pathways to achieve a significant inhibition of tumor growth, especially in EGFR-TKI resistant NSCLC.

It was also observed that the synergistic effects of the dual inhibition of upstream signal EGFR/MET and downstream signal MEK/PI3K on the NSCLC were profound regarding growth inhibition, apoptosis, cell cycle arrest, and expression of phosphorylated pathways. However, the observed effects differed among the three NSCLC cell lines tested depending on their genetic alterations. The H1975 cells were mostly sensitive to the BIBW2992 and ARQ197 combination, and H460 cells to the MEK162/BKM120 combination. The rational explanation might be that the H1975 cells were addicted to EGFR (T790M) and PIK3CA mutation and MET amplification, and H460 cells were addicted to K-RAS and PIK3CA mutation. In a previous study, we demonstrated that the BIBW2992 and ARQ197 combination downregulated the expression of phosphorylated ERK and AKT in H1975 cells [22]. Moreover, as the BIBW2992/ARQ197 combination was cytotoxic, it was observed that the treatment of H1975 cells with this combination exhibited little effect in the decreased phosphorylation of ERK and AKT in comparison to the treatment with the MEK162/BKM120 combination. The study speculated that other signaling pathways that serve downstream of EGFR T790M mutation and MET amplification whose contribution to malignant proliferation are larger than MEK/PI3K. Indeed, the network of signal transduction pathways in NSCLC cells is highly sophisticated. This warrants transcriptome and proteomic profiling under varied physiological conditions to elucidate the exact mechanisms of the mode of action of the therapeutics employed. Therefore, the observed effects of the MEK/PI3K inhibition necessitate further evaluation. In this study, the cytotoxicity of MEK162 combined with BKM120 was confirmed by MTT assay, cell cycle detection and Annexin V-PI double staining detection. Annexin V-PI double staining is a recognized method for detecting apoptosis, especially early apoptosis, with high accuracy. After the cytotoxicity of MEK162 combined with BKM120 on a first-generation EGFR-TKI resistant NSCLC cell line was confirmed, our next study focused on analyzing the effect of the drug on the cell signal transduction pathway, rather than on the expression of apoptotic effector molecules. Therefore, the expression of apoptotic effector molecules such as caspase was not detected. Of course, if time, energy and funds allow, we can carry out related work in the follow-up study to enrich the research results.

The innovation points of this study are as follows: (1) NSCLC cell lines resistant to first-generation EGFR-TKI with different genetic mutation background were used as the research subjects to compare the differences in cytotoxicity, apoptosis and expression of important transduction signal factors between combined inhibition of upstream pathway (EGFR/MET) and combined inhibition of downstream pathway (MEK/PI3K). To increase the basis for preclinical studies on the problem of choosing the combined inhibition of upstream signals located in the cell membrane or the combined inhibition of downstream signals located in the cell after the acquired drug resistance of molecular targeted therapy of NSCLC. This is the most important innovation of this study. (2) At the time of this study (2019), there were more MEK/PI3K inhibitors, but few studies have revealed which agent is less clinically toxic, and the corresponding clinical studies are also few. On the basis of reading a lot of literature, we chose MEK162 and BKM120 as the drugs to be used in the study, mainly because of their good effects and low toxicity. In fact, now more and more evidence shows that our judgment at that time is correct, and clinical studies on MEK162 and BKM120 are also increasing gradually. It shows a good application prospect, especially for MEK162. Our results add to the evidence of efficacy in preclinical studies of these drugs.

Figures 4 and 6 highlight the role of combined inhibition of the upstream pathway EGFR/MET and the downstream pathway MEK/PI3K in each cell line. Both our preliminary experiments and a large number of published papers have confirmed that monotherapy is ineffective in these cell lines, and it has been shown in Figs. 1 and 2 that monotherapy of BKM120 or MEK162 is ineffective in all three cell lines. Therefore, both for theoretical deduction and for saving money and energy in practical application, Results and images of monotherapy of the three cell lines were not shown separately.

In conclusion, dual targeting of MEK and PI3K effectively inhibited EGFR-TKI resistant NSCLC proliferation, promoted cell apoptosis, and induced cell cycle arrest. Therefore, the data generated provide a scientific rationale for co-targeting MEK/PI3K signaling as a strategy for EGFR-TKI resistant NSCLC, especially in K-RAS and PIK3CA mutation in NSCLC.

## Data Availability

We declared that materials described in the manuscript, including all relevant raw data, will be freely available to any scientist wishing to use them for non-commercial purposes, without breaching participant confidentiality. If anyone wish to obtain the study data, please contact the corresponding author: Zhi-Jian Zhang.

## Abbreviations

NSCLC: Non-small cell lung carcinoma
EGFR: Epidermal growth factor receptor
TKIs: Tyrosine kinase inhibitors
MEK: MAP kinase-ERK kinase
ERK: Extracellular-signal-regulated kinase
PKB/AKT: PI3K/protein kinase B
mTOR: Mammalian target of rapamycin
PI: Propidium iodide
PBS: Phosphate-buffered solution
SD: Standard deviation
LSD: Least significant difference
SPSS: Statistics Package for Social Science
TSC1: Tuberous sclerosis protein complex 1
FOXO: Forkhead box O
mTORC1: MTOR complex 1
